# The circular RNA landscape in specific peripheral blood mononuclear cells of critically ill patients with sepsis

**DOI:** 10.1186/s13054-020-03146-4

**Published:** 2020-07-13

**Authors:** Hina N. Khan, Xanthe Brands, Simona Aufiero, Arie J. Hoogendijk, Augustijn M. Klarenbeek, Tjitske S. R. van Engelen, Bastiaan W. Haak, Lonneke A. van Vught, Janneke Horn, Marcus J. Schultz, Aeilko H. Zwinderman, Tom van der Poll, Brendon P. Scicluna

**Affiliations:** 1 Center for Experimental Molecular Medicine, Amsterdam University Medical Centers, location Academic Medical Center, University of Amsterdam, Room G2-105, Meibergdreef 9, 1105 AZ Amsterdam, the Netherlands; 2 Department of Clinical Epidemiology, Biostatistics and Bioinformatics, Amsterdam University Medical Centers, location Academic Medical Center, University of Amsterdam, Amsterdam, the Netherlands; 3 Department of Intensive Care Medicine, Amsterdam University Medical Centers, location Academic Medical Center, University of Amsterdam, Amsterdam, the Netherlands; 4 Mahidol Oxford Tropical Medicine Research Unit, Bangkok, Thailand; 5 Division of Infectious Diseases, Amsterdam University Medical Centers, location Academic Medical Center, University of Amsterdam, Amsterdam, the Netherlands

**Keywords:** Circular RNA, Peripheral blood mononuclear cells, Community-acquired pneumonia, Sepsis

## Abstract

**Background:**

Dysregulation of the host immune response is a pathognomonic feature of sepsis. Abnormal physiological conditions are understood to shift efficient linear splicing of protein-coding RNA towards non-canonical splicing, characterized by the accumulation of non-coding circularized (circ)RNA. CircRNAs remain unexplored in specific peripheral blood mononuclear cells (PBMCs) during sepsis. We here sought to identify and characterize circRNA expression in specific PBMCs of patients with sepsis due to community-acquired pneumonia (CAP) relative to healthy subjects.

**Methods:**

The study comprised a discovery cohort of six critically ill patients diagnosed with sepsis due to community-acquired pneumonia and four (age, gender matched) healthy subjects. PBMCs were isolated, and fluorescence-activated cell sorting was used to purify CD14+ monocytes, CD4+, CD8+ T cells, and CD19+ B cells for RNA sequencing. CD14+ monocytes from independent six healthy volunteers were purified, and total RNA was treated with or without RNase R.

**Results:**

RNA sequencing of sorted CD14+ monocytes, CD4+, CD8+ T cells, and CD19+ B cells from CAP patients and healthy subjects identified various circRNAs with predominantly cell-specific expression patterns. CircRNAs were expressed to a larger extent in monocytes than in CD4+, CD8+ T cells, or B cells. Cells from CAP patients produced significantly higher levels of circRNA as compared to healthy subjects. Considering adjusted *p* values, circVCAN (chr5:83519349-83522309) and circCHD2 (chr15:93000512-93014909) levels in monocytes were significantly altered in sepsis. Functional inference per cell-type uncovered pathways mainly attuned to cell proliferation and cytokine production. In addition, our data does not support a role for these circRNAs in microRNA sequestration. Quantitative PCR analysis in purified monocytes from an independent group of healthy volunteers confirmed the existence of circVCAN and circCHD2.

**Conclusions:**

We provide a benchmark map of circRNA expression dynamics in specific immune cell subsets of sepsis patients secondary to CAP. CircRNAs were more abundant in immune cells of sepsis patients relative to healthy subjects. Further studies evaluating circRNA expression in larger cohorts of sepsis patients are warranted.

## Background

Sepsis is a complex syndrome initiated by an imbalanced reaction to infection, with community-acquired pneumonia (CAP) as a major cause, leading to organ damage and high mortality rates [1, 2]. Despite advances in critical care medicine and antimicrobial therapy, no drug specifically targeting sepsis has been approved [3]. The immunopathology of sepsis is understood to encompass a plethora of host defense reactions that are primarily categorized as either abnormally excessive or suppressed [4]. Excessive inflammation (or hyperinflammation) exacerbates organ dysfunction whereas immune suppression induces immune cells to become perpetually unresponsive to pathogens, thereby increasing the risk of mortality due to, for example, uncontrolled secondary infections and failure to restore homeostasis [5]. Prospective observational cohorts to study the genomics of sepsis have shown that patients present signatures of both sustained inflammation and immune suppression [6–8]. Identifying regulatory features of the genomic response in sepsis is crucial for future drug discovery.

Advances in next-generation sequencing and bioinformatics have aided in the identification of a novel type of RNA class that is naturally resistant to degradation by exonucleases, termed circular (circ)RNA [9]. CircRNAs are single-stranded RNA molecules formed by non-canonical pre-messenger (m) RNA splicing, where downstream donor sites are “back-spliced” to upstream acceptor sites (e.g., the end of exon 4 covalently binds to the start of exon 4) [10]. In general, the abundance of circRNA is low when compared to the associated linear mRNA [11–13], suggesting that the formation of circRNA may be inefficient for cellular physiology [14]. While the functions of circRNA remain largely unknown, proposed roles include sequestration of microRNA, protein binding, and interference in transcript splicing [15]. The patterns of circRNA expression in peripheral blood mononuclear cells (PBMCs) of critically ill patients with sepsis remain unexplored. Here, we sought to characterize the circRNAome in specific PBMCs of sepsis patients secondary to CAP relative to healthy subjects. Our findings provide a benchmark in understanding the role of circRNA in the genomic response during sepsis secondary to CAP.

## Methods

### Study population

The study comprised a discovery cohort of six critically ill patients with sepsis due to community-acquired pneumonia (CAP) with positive blood cultures for *Streptococcus pneumoniae* (*S. pneu*) selected from within the framework of the Molecular diAgnosis and Risk stratification in Sepsis (MARS) project (ClinicalTrials.gov identifier NCT01905033) [16, 17]. Sepsis diagnosis was described in detail previously [16, 18]. This cohort was enrolled between January 2011 and July 2012. The Medical Ethics committees approved an opt-out consent method (IRB no. 10-056C). Severity was assessed by Acute Physiology and Chronic Health Evaluation (APACHE) IV [19] and total Sequential Organ Failure Assessment (SOFA) scores excluding the central nervous system component [20]. Shock was qualified by the use of vasopressors (norepinephrine, epinephrine, or dopamine) for hypotension in a norepinephrine-equivalent dose of more than 0.1 μg/kg/min [2]. Four healthy subjects (age and gender matched) were also included in the discovery cohort. A total of six independent healthy volunteers (> 18 years old) were also included for in vitro validation assays. From all healthy subjects, written informed consent was obtained.

### Peripheral blood mononuclear cell isolation

Heparinized whole blood (10 ml) was collected from healthy subjects and sepsis patients. Blood from sepsis patients was collected within 24 h of ICU admission and diluted 1:1 with phosphate-buffered saline (PBS) (Fresenius Kabi). PBMCs were separated by centrifugation using Ficoll-Paque PLUS (GE Healthcare Life science, Little Chalfont, UK) followed by treatment with ice-cold erythrocyte lysis buffer (Qiagen, Venlo, The Netherlands) and washed twice with ice-cold PBS supplemented with 0.5% sterile endotoxin-free bovine serum albumin (BSA; Divbio Science Europe, AK8917-0100). PBMCs were stained with cluster of differentiation (CD)14-APC-Cy7, CD3-AlexaFluor700, CD4-PERCP-Cy5.5, CD15-FITC, CD20-PE-CF594 (BD Biosciences), and CD8-APC (eBioscience, San Diego, CA) for 30 min at 4 °C. Within a lymphocyte gate based on forward and side scatter, CD4 T cells were defined as CD3^+^CD4^+^CD8^−^, CD8 T cells as CD3^+^CD8^+^CD4^−^, and B cells as CD3^−^CD19^+^. In the monocyte gate, monocytes were identified as CD14^+^CD15^−^ cells. Data acquisition was performed using a FACSCanto II flow cytometer (BD Biosciences). After sorting, cells were kept in RNAprotect Cell reagent (Qiagen, Venlo, The Netherlands #76526). Monocytes were also captured from six healthy subjects enrolled for in vitro validation assays. Healthy PBMCs were purified from heparinized whole blood using Ficoll-Paque as described above, and monocytes were purified with magnetic beads coated with anti-CD14 antibody respectively (Miltenyi Biotech). Monocyte purity was verified by flow cytometry (> 95% CD14^+^CD15^−^ cells). Flow cytometry data were analyzed with Flowjo X.07 (Tree Star, Ashland, OR).

### RNA sequencing

Total RNA was isolated from each cell type by means of the miRNeasy RNA isolation kit (Qiagen, Venlo, The Netherlands) according to the manufacturer’s instructions. RNA quality was assessed by bioanalysis (Agilent), with all samples having RNA integrity numbers (RINs) > 7. Total RNA concentrations were determined by Qubit® 2.0 Fluorometer (Life Technologies, Carlsbad, CA, USA). Sequencing libraries were prepared by means of the Ovation® RNA-Seq System V2 (NuGEN) kit as per the manufacturer’s instructions. Libraries were sequenced using the Illumina HiSeq2500 (Illumina) to generate 2 × 126 bp paired-end (PE) reads. Sequence read quality was assessed by means of the FastQC methods (version 0.11.5; Babraham Institute, Babraham, Cambridgeshire, UK). Trimmomatic version 0.32 [21] was used to trim the Illumina adapters containing poor-quality bases and ambiguous nucleotide containing sequences. Low-quality leading (3 nucleotides) and trailing (3 nucleotides) bases were removed from each read, and the quality of the body of the reads was assessed with a sliding window trimming using a window of 4 and a phred score threshold of 15 nucleotides. After pre-processing, the remaining high-quality reads were used to align against the Genome Reference Consortium Human Genome Build 38 patch release 7 (GRCh38.p7) [22]. Mapping was performed by Tophat2 version 2.1.1 [23] with parameters as default. Count data were generated by means of the featureCounts method [24] and analyzed using the DESeq2 method [25] in the R statistical computing environment (R Core Team 2014. R: A language and environment for statistical computing. R Foundation for Statistical Computing, Vienna, Austria). Significance was defined by Benjamini-Hochberg (BH) adjusted *p* value ≤ 0.05 and fold change ≥ 1.5 or ≤ − 1.5. The bioinformatics workflow is represented in Additional file 1: Fig. S1.

### Circular RNA bioinformatics

Sequence reads were analyzed by Mapsplice2 [26] with the following parameters: --fusion-non-canonical, --min-fusion-distance 200, and --min-map-len 25. The short read aligner Bowtie [27] was used to align the reads to the reference genome (GRCh38.p7). A circRNA was called if it was supported by at least four back-spliced reads in at least two different samples. To perform reverse complementary sequence (RCS) analysis, we aligned the downstream and reversed complement of the upstream intron using the pairwiseAlignment function implemented in the Biostrings R package [28]. The identified significant [29] RCSs were then blasted using the RepeatMasker program [30] to screen sequences for interspersed repeats and low complexity DNA sequences. The flanking intron sequences of all circRNAs were obtained from the GENCODEv25.p7 human genome reference. The RNA-hybrid tool [31] was used to predict putative micro (mi) RNA target sites in circRNA. To determine the relative expression of circRNA with respect to the host linear RNA, we used the back-splice-to-linear ratio as described previously [32], modified by taking the average of read counts for all samples (*S*1…*Sn*).

$$ \mathrm{Back}-\mathrm{splice}-\mathrm{to}-{\mathrm{linear}\ \mathrm{ratio}}_{\left(\mathrm{Gene}\right)}=\frac{\mathrm{Average}\left({c}_{\left(S1\dots Sn\right)}\right)}{\operatorname{Max}\left(\mathrm{Average}\left(l{1}_{\left(S1\dots Sn\right)}\right),\mathrm{Average}\left(l{2}_{\left(S1\dots Sn\right)}\right)\right)} $$

where *c* is total read count of circRNA back-splice junction and *l*1 and *l*2 represent the total linear read count that spans left and right linear-spliced junctions of the same exon(s), respectively.

For miRNA enrichment analysis, a cumulative distribution function (CDF) was computed using the ecdf function of the R Bioconductor package, Stats. The University of California Santa Cruz (UCSC) table browser [33] and data integrator tool along with custom tracks were used to obtain the coordinates and exon sequences. miRbase (release 22) was used to get the human mature miRNA sequences. Pathway analysis of the circRNA host genes was done by gene ontology (GO) enrichment analysis [34] and Ingenuity pathway analysis (IPA; Qiagen bioinformatics). Human species and IPA gene knowledgebase were selected. All other settings were default. BH multiple-test adjusted *p* values < 0.05 defined significance.

### Validation assay

CD14+ monocytes purified from six healthy volunteers were seeded at 0.5 × 10^6^ cells per well with Roswell Park Memorial Institute (RPMI) medium supplemented with 10% sterile fetal calf serum (FCS; HyClone, #SV30160.03), 200 mM glutamax (Thermo Fisher, #35050-038), 100 μM pyruvate (Thermo Fisher, #11360-039), and 50 μg/ml gentamycin (Lonza, #17-5192) in a cell-repellent surface 48-well plate (Greiner Bio-one, #677970). Total RNA was isolated from purified monocytes using the RNeasy Mini Kit (Qiagen, #74104) according to the manufacturer’s instructions. RNA quality and concentration were assessed using Nanodrop (Thermo Fisher). To generate RNase R digested RNA [35], 150 ng total RNA was incubated in 1x RNase R buffer in a 20-μl reaction with or without 5 units of RNAse R (Epicentre) at 37 °C for 10 min followed by heat inactivation at 95 °C for 3 min. DNA was depleted using DNase I (Invitrogen, #79254). Complementary DNA (cDNA) was synthesized with random primers using the SuperScript III reverse transcriptase (RT) kit (Invitrogen; #11752250) as per the manufacturer’s instructions. Divergent primers were designed for the versican (*VCAN*; chromosome (chr)5: 83519349-83522309) and chromodomain helicase DNA binding protein 2 (*CHD2*; chr15: 93000512-93014909) loci (Additional file 2: Fig. S2). The sequences of *VCAN* circRNA primers were 5′-GCCCCCAGCAAGCACAAAATTT-3′ (forward) and 5′-TGCAGTTTCTGCGAGGATACTC-3′ (reverse). The sequences of the *CHD2* circRNA primers were 5′-TCACCCCAACAAGAGACACTTC-3′ (forward) and 5′-TCTTTCAGCCTGGGCACTTTGT-3′ (reverse). The hypoxanthine phosphoribosyltransferase (*HPRT*) gene was used as linear messenger (m) RNA control. The sequences of the *HPRT* primers were 5′-GGATTTGAAATTCCAGACAAGTTT-3′ (forward) and 5′-GCGATGTCAATAGGACTCCAG-3′ (reverse). Quantitative reverse-transcriptase polymerase chain reaction (qRT-PCR) was performed by using the SensiFAST SYBR No-ROX Mix (Bioline, #CSA-01190) and a LightCycler480 system II (Roche) using the following program: 95 °C pre-incubation for 6 min followed by 40 cycles of 95 °C (10 s), 62 °C (20 s), and 72 °C (20 s). RT-PCR products were separated by agarose gel (2%, Roche #11388991001) electrophoresis, and bands were visualized by Syngene Gbox scanner. Data were quantified and analyzed by means of the LinRegPCR method [36]. CircRNA expression indices of treated samples were normalized to corresponding linear *HPRT1* expression in untreated samples.

### Monocyte stimulation

In order to evaluate the inducibility of circRNA, monocytes from healthy participants were stimulated with lipopolysaccharide (LPS; EB Ultrapure Invivogen #tlrl-3pelps), heat-killed S*treptococcus* (S.) *pneumoniae* (Sp; ATCC6303), and *Klebsiella* (K.) *pneumoniae* (Kp; ATCC43816) at 37 °C with 5% CO_2_ and 95% humidity for 2 and 24 h. Heat-killed bacteria (70 °C for 30 min) were used at a bacteria-to-cell ratio of 10:1. Supernatants were collected for enzyme-linked immunosorbent assays (ELISAs). Tumor necrosis factor alpha (TNF) and interleukin (IL)-6 levels were measured using commercially available ELISA kits (TNF, R&D systems #MAB610; IL-6, Thermo Fisher #88710677). Bradford protein assay (BIO-RAD, Hercules, CA) was used for total protein measurements. Cells were resuspended in RNAprotect Cell reagent (Qiagen, Venlo, The Netherlands #76526) and stored at − 80 °C prior to qRT-PCR analysis.

### Statistics

The length of exons involved in circRNAs, introns flanking the expressed circRNAs, and the control set were compared using an unpaired Student *t* test. The paired Student *t* test was used to compare the circRNA expression with its linear counterpart. Quantitative RT-PCR data was analyzed by Wilcoxon rank-sum test. Unless otherwise stated, *p* < 0.05 demarcated significance. Heat maps were generated using the pheatmap R method, and principal component analysis (PCA) was done using the FactoMinerR R package. Venn diagrams were made using the VennDiagram R package.

## Results

### Circular RNA detection and characterization in CAP and health

**Fig. 1:**
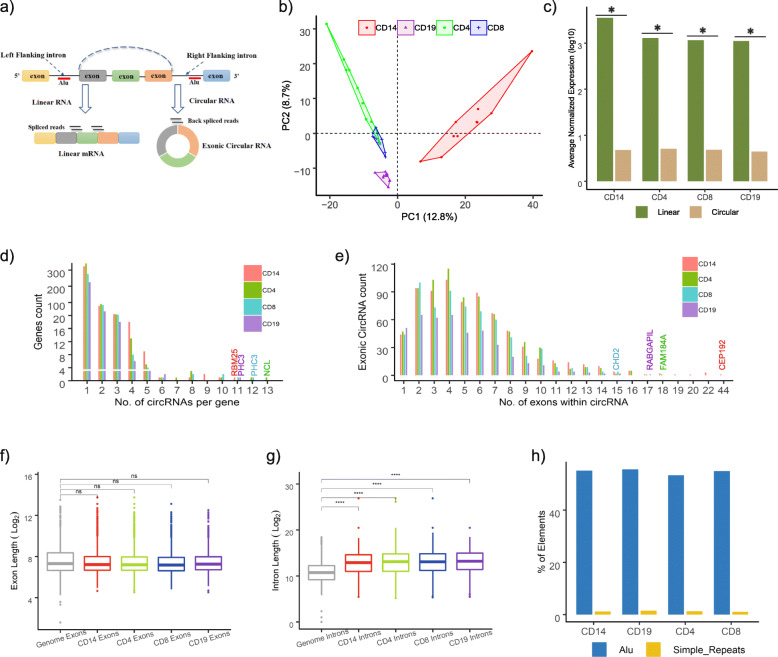
Detection and characterization of circRNA in monocytes (CD14), CD4, CD8 T cells, and B cells (CD19) of CAP patients and healthy subjects. **a** Schematic illustration of linear RNA splicing and the back-spliced junction-read leading to the identity of exonic circRNA. Alu, *Arthrobacter luteus* restriction endonuclease repeats. **b** Principal component analysis (PCA) plot of all detected circRNAs for monocytes, CD4, CD8 T cells, and B cells (CD19). **c** Linear RNA and circRNA expression indices for each peripheral mononuclear cell type. **d** Number of detected circRNAs per gene in monocytes, CD4+, CD8+ T cells, and B cells. **e** Number of circRNAs that contained different number of exons derived from parental genes in monocytes, CD4, CD8 T cells, and B cells. **f** Box plot showing log2-transformed (log2) exon length of back-spliced exons in each cell type relative to the genome-wide exon length. **g** Box plot depicting log2-transformed intron lengths of introns flanking circRNA back-spliced exons for monocytes (CD14), CD4, CD8 T cells, and B cells (CD19) and genome-wide lengths. **h** Barplot showing the percentage of repetitive elements in introns flanking circRNAs of each cell type. Alu, *Arthrobacter luteus* restriction endonuclease repeats

RNA-sequencing data of monocytes, CD4^+^, CD8^+^ T cells, and B cells isolated from healthy subjects (median age [Q1–Q3], 54 [50–60]; males [%], 2 [50]) and CAP patients were used to map circRNA (Fig. 1a). The CAP patients consisted of elderly adults (median age [Q1–Q3], 62 [50–68]; males [%], 1 [17]) with median APACHE IV score of 52 (Q1–Q3, 47–74), median SOFA score of 6 (Q1–Q3, 5–8), 50% having shock, and 67% on mechanical ventilation. In total, 734, 753, 636, and 430 circRNAs were identified in monocytes, CD4+, CD8+ T cells, and B cells, respectively **(**Additional file 3: Table S1). PCA of circRNA expression revealed clearly distinct clusters per cell type with explainable variance of 12.8% (PC1) and 8.7% (PC2) (Fig. 1b). On average, circRNAs were expressed to a significantly lower extent than the corresponding host gene expression (Fig. 1c). Monocytes produced more circRNA than CD4+, CD8+ T cells, or B cells, with back-splice-to-linear ratios (a measure of circRNA expression relative to linear mRNA counterpart) equating to 0.13 (Q1–Q3, 0.05–0.39), 0.06 (Q1–Q3, 0.05–0.26), 0.07 (Q1–Q3, 0.06–0.29), and 0.05 (Q1–Q3, 0.02–0.06), respectively. The gene encoding nucleolin (*NCL*) produced the most circRNA species (total of 13), polyhomeotic homolog 3 (*PHC3*) produced 12 circRNAs, and RNA binding motif protein 25 (*RBM25*) produced 11 circRNAs (Fig. 1d). The longest predicted circRNA encompassed 44 exons of the centrosomal protein 192 (*CEP192*) gene (chr18: 12999421-13117643) (Fig. 1e). Of note, while the average length of circRNA exons was not longer than the genome-wide average (Fig. 1f), flanking introns were significantly longer (Fig. 1g). Furthermore, the flanking introns of the predicted circRNAs were significantly enriched for RCSs, containing interspersed *Arthrobacter luteus* (Alu) restriction endonuclease repeats (55%) and simple repeats (1.3%) (Fig. 1h). These findings are consistent with genomic features that convey RNA circularization [13–15]. Altogether, circRNAs were produced by each immune cell type in both healthy individuals and CAP patients. Monocytes were predicted to produce substantially more circRNAs than T or B cells.

### Circular RNA expression patterns in CAP relative to health

**Fig. 2:**
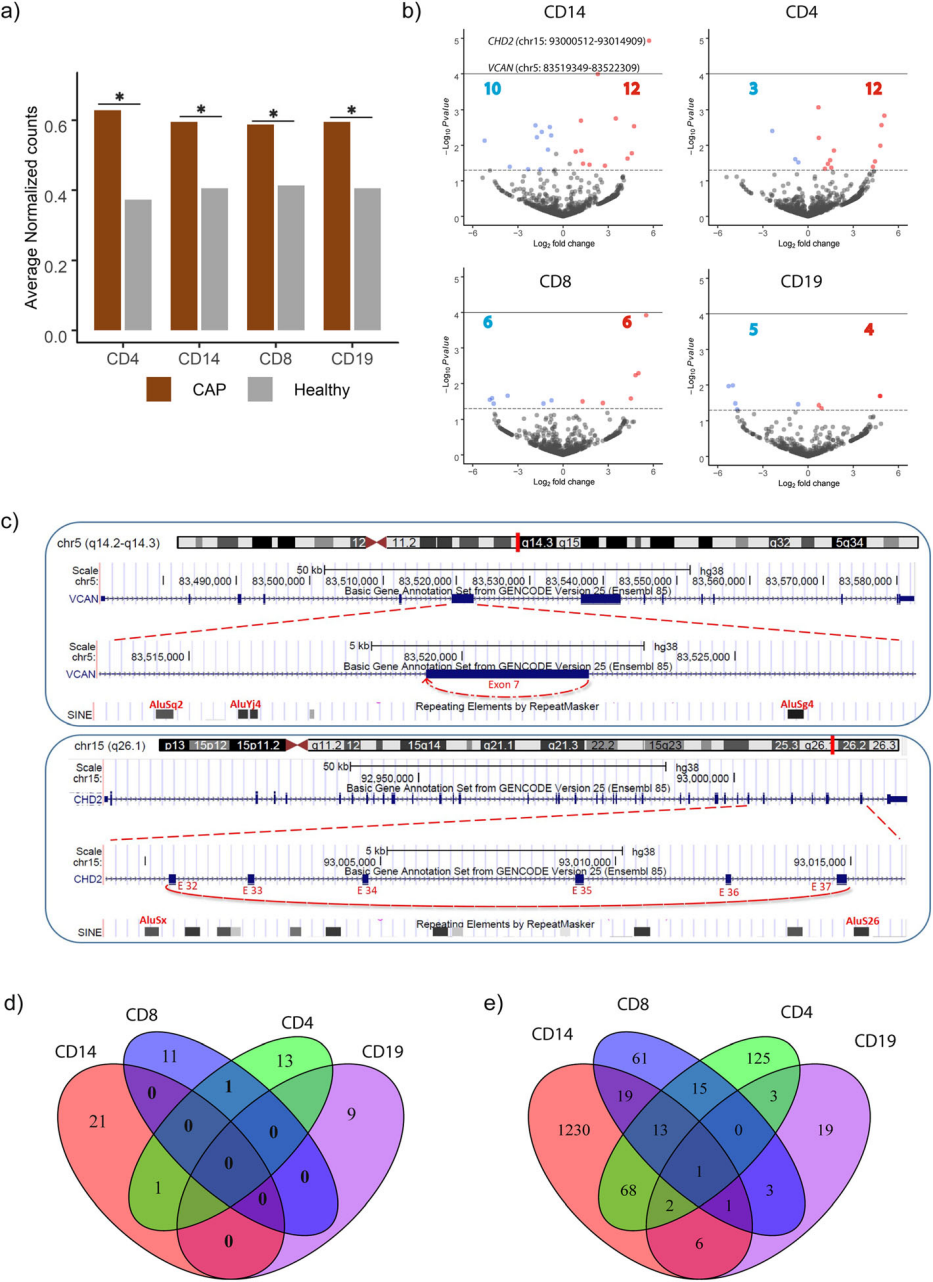
Circular RNA expression in patients with sepsis due to CAP relative to healthy subjects. **a** Barplot illustrating the average circRNA expression in monocytes (CD14), CD4, CD8 T cells, and B cells (CD19) of CAP patients and healthy volunteers. **b** Volcano plot representing the differentially expressed circRNA of each peripheral mononuclear cell type in CAP as compared to the health. The horizontal dotted line represents the nominal *p* value ≤ 0.05 threshold. A horizontal solid line represents the multiple-comparison adjusted *p* value ≤ 0.05 threshold. Red dots, upregulated circRNA (nominal *p* < 0.05); blue dots, downregulated circRNA (nominal *p* < 0.05); gray dots, not altered circRNA (nominal *p* > 0.05). **c** Representative UCSC genome browser of the exons corresponding to the two significantly altered (adjusted *p* value < 0.05) circRNAs in monocytes of CAP patients relative to healthy patients. The presence of Alu repeats in the flanking introns is noted. E, exon; Alu, *Arthrobacter luteus* restriction endonuclease repeats. **d** Venn diagram of differentially abundant circRNAs (nominal *p* < 0.05) per cell type. **e** Venn diagram of differentially expressed (adjusted *p* < 0.05) linear mRNA species in monocytes, CD4, CD8 T cells, and B cells (CD19) of CAP patients relative to healthy subjects

Here, we sought to identify significantly altered circRNA expression in monocytes, T cells, and B cells of CAP patients relative to healthy samples. In general, cells of CAP patients produced more circRNA than healthy participants (Fig. 2a). Considering multiple-comparison adjusted *p* values, circRNAs from the versican gene (*VCAN*; chr5: 83519349-83522309) and chromodomain helicase DNA binding protein 2 gene (*CHD2*; chr15: 93000512-93014909) were significantly more abundant in monocytes of CAP patients (Fig. 2b, c). A list of detected and altered circRNA can be found in Additional file 3: Table S1. CircRNA expression differences between study groups were largely unique to cell type, and none showed constitutive alterations in all cells (Fig. 2d). This observation was also evident for linear RNA species with predominantly cell-type-specific expression patterns in CAP patients compared to healthy subjects (Fig. 2e and Additional file 4: Fig. S3). Of the 1340, 227, 113, and 35 significantly altered genes (adjusted *p* < 0.05) in monocytes, CD4+, CD8+ T cells, and B cells, respectively (Additional file 4: Fig. S3), 1230, 125, 61, and 19 genes were specifically altered in respective cell types (Fig. 2e). Notably, *VCAN* expression in monocytes was significantly higher in CAP relative to health (adjusted *p* = 0.0013). No differences were detected for *CHD2* gene expression. Our findings showed largely cell-type-specific circRNA expression patterns and specific circRNA were significantly altered in monocytes of CAP patients relative to healthy subjects.

### Functional inference of circular RNA

**Fig. 3:**
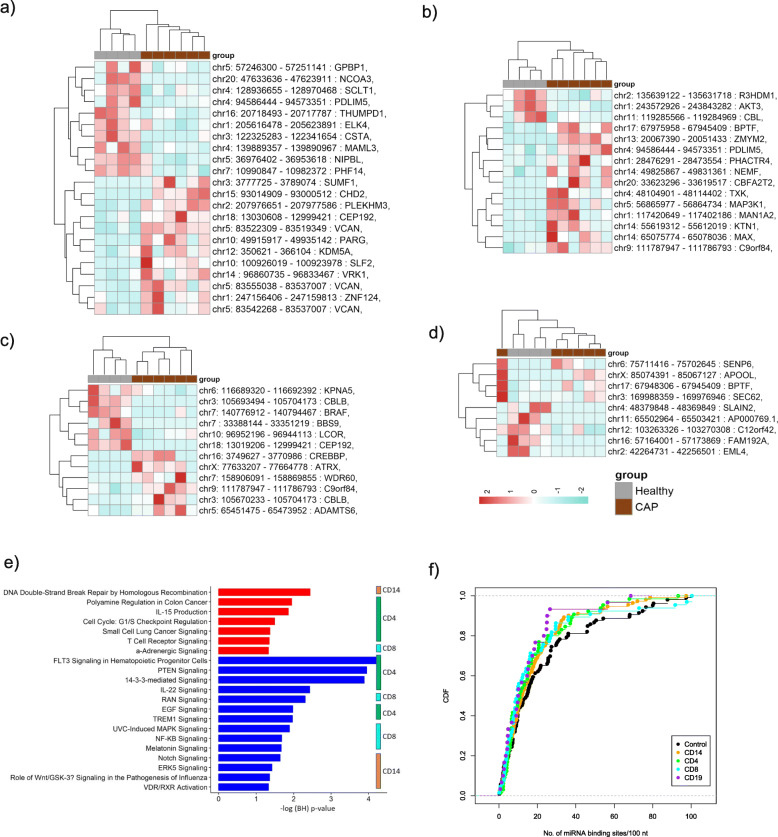
Functional inference of circRNAs identified in monocytes (CD14), CD4, CD8 T cells, and B cells (CD19) of CAP patients and healthy subjects. **a**–**d** Heatmaps showing expression patterns of differentially altered circRNAs (nominal *p* value < 0.05) in monocytes (**a**), CD4+ T cells (**b**), CD8+ T cells (**c**), and B cells (**d**) of CAP patients relative to healthy subjects. **e** Pathway analysis of the host genes producing the circRNAs for monocytes and CD4+, CD8+ T cells. The vertical axis represents the pathways, and the horizontal axis represents the -log10-transformed Benjamini-Hochberg (BH) adjusted *p* values. Red bars represent pathways for host genes of upregulated circRNA, whereas blue bars denote pathways for host genes of downregulated circRNA in CAP relative to health. Vertical bars depict the cell type, that is, monocytes (orange), CD4+ (green), and CD8+ (cyan). **f** Cumulative distribution function (CDF) showing the abundance of predicted micro (mi) RNA binding in the exons of differentially abundant circRNAs of monocytes (orange), CD4+ (green), CD8+ (cyan) T cells, and B cells (purple) and human host gene exons that do not circularize (controls, black)

In order to infer on putative functions and potential biological roles of the altered circRNA identified in each cell type, we firstly evaluated cellular biological pathway enrichment of the “host” genes producing the circRNAs. Genes producing circRNAs that were differentially abundant in monocytes, CD4+, and CD8+ T cells of CAP patients (relative to healthy subjects) (Fig. 3a–c) were associated to distinct canonical signaling pathways (Fig. 3d). In particular, monocyte circRNA was primarily associated with Notch signaling and ERK signaling pathways. Numerous pathways were detected for low expression circRNA in CD4+ T cells, including FLT3 signaling in hematopoietic progenitor cells (Fig. 3d), as well as RAN signaling and UVC-induced MAPK signaling for CD8+ T cells. High expression circRNA was mainly associated with IL-15 production and DNA double-strand break repair by homologous recombination pathways (Fig. 3d). Secondly, motivated by reported functions of circRNA as miRNA “sponges” [13], we explored circRNA sequences for potential miRNA binding regions. The frequency of miRNA binding sites in circRNA exons was not different to all other exons in the genome that did not produce circRNA (Fig. 3e). Therefore, the lack of miRNA binding site enrichment in the herein predicted circRNA does not support their role as miRNA “sponges”.

### Experimental validation

**Fig. 4:**
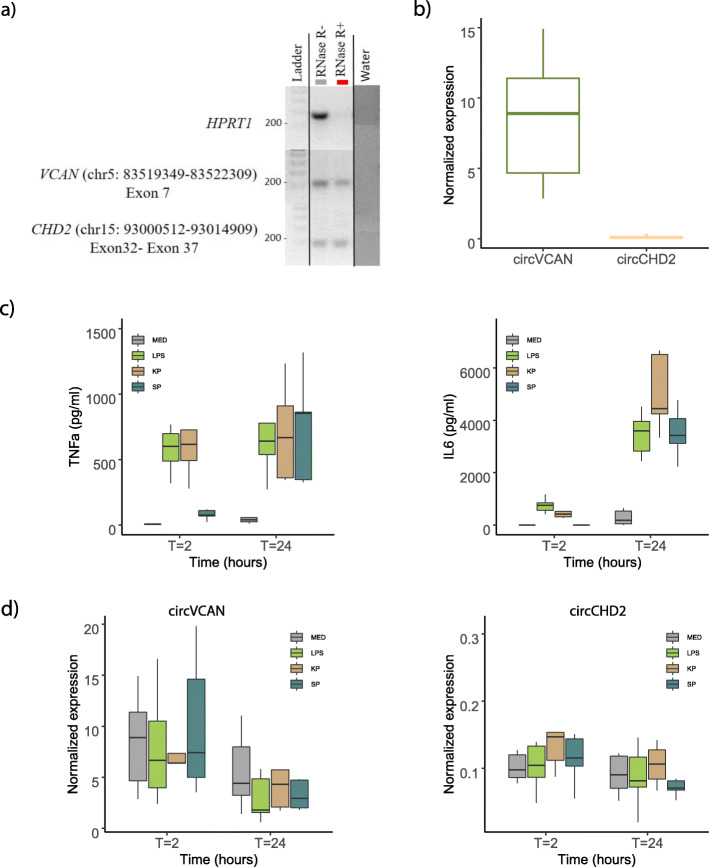
Experimental validation of detected circRNAs in healthy CD14+ monocytes. **a** Agarose gel scan illustrating amplicons of linear mRNA (*HPRT1*) and two circRNAs *VCAN* (chr5: 83519349-83522309) and *CHD2* (chr15: 93000512-93014909) treated with (+) or without (−) RNase R. Ladder, Thermo Scientific GeneRuler DNA Ladder mix #SM0333. **b** Quantitative RT-PCR normalized expression patterns of *VCAN* (chr5: 83519349-83522309) and *CHD2* (chr15: 93000512-93014909) circRNAs in healthy monocytes. **c** Box plots depicting the production of TNF and IL-6 by monocytes stimulated with lipopolysaccharide (LPS), heat-killed *Streptococcus pneumoniae* (Sp), and *Klebsiella pneumoniae* (Kp) for 2 and 24 h. *p < 0.05. **d** Box plots illustrating the normalized expression patterns of circRNAs, circVCAN (chr5: 83519349-83522309) and circCHD2 (chr15: 93000512-93014909), in stimulated monocytes

Thus far, we have identified putative circRNA species, their expression patterns, and potential functional pathways in different peripheral blood mononuclear cell types from CAP patients and healthy volunteers. Here, we sought to provide robustness to our bioinformatics approach by experimental validation of circRNA existence in an independent population of healthy subjects. We focused our attention on the two significantly altered circRNAs in monocytes, that is, *VCAN* (chr5: 83519349-83522309; circVCAN) and *CHD2* (chr15: 93000512-93014909; circCHD2). Prior to quantitative RT-PCR analysis, we treated total RNA from monocytes with or without the RNA digestion enzyme, RNase R. As expected, treatment with RNase R resulted in a reduction in *HPRT1* transcripts (non-circular endogenous control) (Fig. 4a). In contrast, no reduction was observed for circVCAN and circCHD2 (Fig. 4a), which indicated resistance to RNase digestion. CircVCAN was more abundant than circCHD2 (Fig. 4b). These results provide robustness to the existence of circVCAN and circCHD2 in human monocytes.

Next, we evaluated the inducibility of circVCAN and circCHD2 by stimulating monocytes with LPS, as well as clinically relevant *K. pneumoniae* and *S. pneumoniae*. As expected, TNF and IL-6 protein levels in the supernatants were significantly elevated after 2- and 24-h stimulation (Fig. 4c). In contrast, circVCAN and circCHD2 were not induced (Fig. 4d). Altogether, our findings provide benchmark evidence of the existence of circVCAN and circCHD2 in healthy human monocytes, which were not inducible by stimulation with LPS nor clinically relevant bacteria.

## Discussion

Transcriptional activity of genes is not exclusive to production of linear mRNA molecules for translation to proteins. A variety of mature RNA species can be produced per gene, including thousands of circRNA. In the current study, we demonstrated the presence and quantified the abundance of circRNAs in specific PBMCs of sepsis patients secondary to CAP relative to healthy subjects. In general, circRNA production was significantly higher in CAP patients relative to healthy patients, and expression patterns were largely specific to either monocytes, CD4^+^, CD8^+^ T cells, or B cells. Functional inference showed potential roles of circRNA in distinct pathways per cell type, including cytokine production and cell differentiation. Moreover, our data does not support a reported role of circRNA in sequestration of miRNA. High-confidence circRNA was detected in monocytes of an independent cohort of healthy volunteers by exoribonuclease RNase R digestion and quantitative RT-PCR. Altogether, our study provides a benchmark in the identification, characterization, and potential functional implications of the circRNAome in important immune cell types of sepsis patients secondary to CAP.

Animal genomes produce thousands of circRNA molecules in complex tissue, cell type, and developmental stage-specific ways [37]. CircRNA represents a novel class of RNA molecules produced by transcriptional “back-splicing” and composed of only exons, exon-introns, or only introns. Contrary to the past notion that circRNA represented by-products of splicing errors, reported functions include transcriptional regulation of protein-coding RNA. For example, circRNAs CDR1as (ciRS-7) and circ-SRY were shown to sequester miRNAs, thereby altering transcriptional outputs [37, 38]. Furthermore, recent studies suggest circRNA may not only function as non-coding transcriptional regulators but may also harbor potential for translation to amino acids, and ultimately proteins [39]. For instance, overexpression of circ-FBXW7 that encodes a novel 21 kDa protein composed of 185 amino acids, named F-Box and WD Repeat Domain Containing 7 (FBXW7), repressed cellular proliferation and glioma tumorigenesis [40]. It is becoming evident that non-canonical transcriptional “back-splicing” can compete with canonical linear splicing that produces mRNA for translation to protein products [41]. In particular, conditions of cellular stress, for example, in drosophila inhibition of splicing machinery, were shown to shift the steady-state output of pre-mRNA splicing towards circularization [42]. Critical illness due to sepsis is associated with severe cellular stress, which oftentimes precipitates to a condition of “immunoparalysis,” thereby making immune cells non-responsive to secondary challenges [4, 7]. The extent of circRNA effects on immune cell responsiveness and tolerance to bacterial ligands (e.g., endotoxin) certainly warrant exploration.

Assigning biological functions to circRNA is an emerging field of functional genomics. The use of bioinformatics tools and molecular biology can elucidate their function in cellular physiology and define their role in disease conditions. The circRNA-miRNA “sponge” paradigm has been well characterized in the literature [43]. Notably, binding of miRNA to circRNA molecules does not necessarily constitute miRNA suppression. CircRNA-miRNA complexes may convey susceptibility to degradation, which in turn release miRNA to the intracellular milieu. This was evidenced in a recent report that showed ciRS-7 bound to miR-671 created an Argonaute 2 cleavage site, which released the “passenger” miR-7 in the process and affecting brain function [44]. Therefore, this suggested that circRNAs may function as miRNA reservoirs and facilitate miRNA transportation [45]. Based on our in silico evaluation of miRNA seed sequences present in exons within circRNA structures relative to those exons that do not form part of circRNA, we found no statistical overrepresentation. This suggests that the altered circRNA levels in monocytes, T cells, and B cells of sepsis patients relative to healthy subjects may partake in cellular functions that are not exclusive to circRNA sponging. Many circRNAs are predicted to harbor RNA binding protein sites [46], with a few reports demonstrating the capacity of circRNA for protein binding. For example, circMBL/MBNL1 generated in both drosophila and humans was demonstrated to specifically and strongly bind to the RNA binding protein muscleblind (MBL/MBNL) [41]. Furthermore, and in line with our findings, circRNA expression was shown to be cell-type-specific [47], which may suggest functionality of circRNA is also dependent on the type of cell and differentiation state. Of note, enucleated blood cells, that is, erythrocytes and platelets, were reported to contain the highest levels of circRNA when compared to all hematopoietic cell lineages [47].

CircRNA detection has been reported to be in part dependent on the detection algorithm. Thus, to ascertain robustness in circRNA detection, it is important to combine bioinformatics and experimental approaches. By leveraging on a fundamental property of circRNA, that is, the lack of a free 3′ end required for digestion with RNase R [13–15], a 3′ to 5′ exoribonuclease enzyme, it is possible to differentiate linear and circRNA species. In this way, we showed that circRNAs *VCAN* (chr5: 83519349-83522309) and *CHD2* (chr15: 93000512-93014909) were detectable in healthy monocytes. While we focused our attention on the two significantly altered circRNAs in CAP compared to health, the presence of other circRNA species in all cell types is certainly not excluded. The limited number of significantly altered circRNA in CAP relative to health can be explained, at least in part, by the low sample size in our discovery phase by RNA-seq. Furthermore, validating circRNA existence and expression in CAP patients would further heighten the robustness of our findings, though the availability of RNA from specific immune cells was limiting. Future studies specifically designed to evaluate circRNA expression and biology in larger cohorts of patients with sepsis are warranted.

## Conclusion

In conclusion, this is the first study to delineate the expression profiles of circRNAs in specific immune cell types of critically ill patients with sepsis secondary to CAP relative to health. Levels of circRNA were significantly elevated in PBMCs of sepsis patients, with monocytes producing the highest amounts of circRNA relative to CD4+, CD8+ T cells, and B cells. CircRNA expression was significantly altered in patients as compared to healthy subjects, with high specificity for the type of mononuclear cell. By detecting the existence and accumulation of circRNAs in important immune cell subsets, our study adds another layer of regulation to the host response. Therefore, we provide a framework that not only offers new insight into the immunopathology of sepsis but may also represent a novel avenue for drug discovery.

## Supplementary information

**Additional file 1: Figure S1.** Workflow of the analysis of the circular RNAs.

**Additional file 2: Figure S2.** Schematic of the divergent primers used for the detection of cicrRNAs.

**Additional file 3: Table S1.** Table of differentially abundant circRNAs.

**Additional file 4: Figure S3.** Linear RNA transcriptional changes in monocytes, CD4+ and CD8+ T-cells and CD19+ B-cells of CAP patients compared to healthy subjects.

## Data Availability

Data are accessible in the National Center for Biotechnology Information Gene Expression Omnibus [48] accession number GSE136200.

## Abbreviations

circRNA: Circular RNA
PBMCs: Peripheral blood mononuclear cells
CAP: Community-acquired pneumonia
RCS: Reverse complementary sequence
miRNA: MicroRNA
RIN: RNA integrity number
PCA: Principal component analysis
