# Supplementary figures and images for: The circular RNA landscape in specific peripheral blood mononuclear cells of critically ill patients with sepsis
Source: Crit Care. 2020 Jul 13;24:423. doi: 10.1186/s13054-020-03146-4 (PMC7359566; doi:10.1186/s13054-020-03146-4)

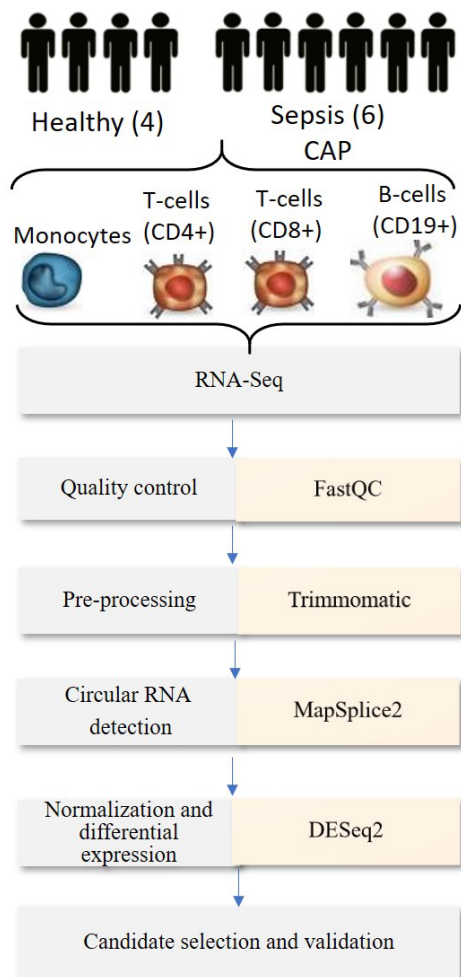

Supplementary Figure 1: Workflow of the analysis of the circular RNAs.

Supplement: Supplementary file 1 — Additional file 1: Figure S1. Workflow of the analysis of the circular RNAs. [file 13054_2020_3146_MOESM1_ESM.pdf]

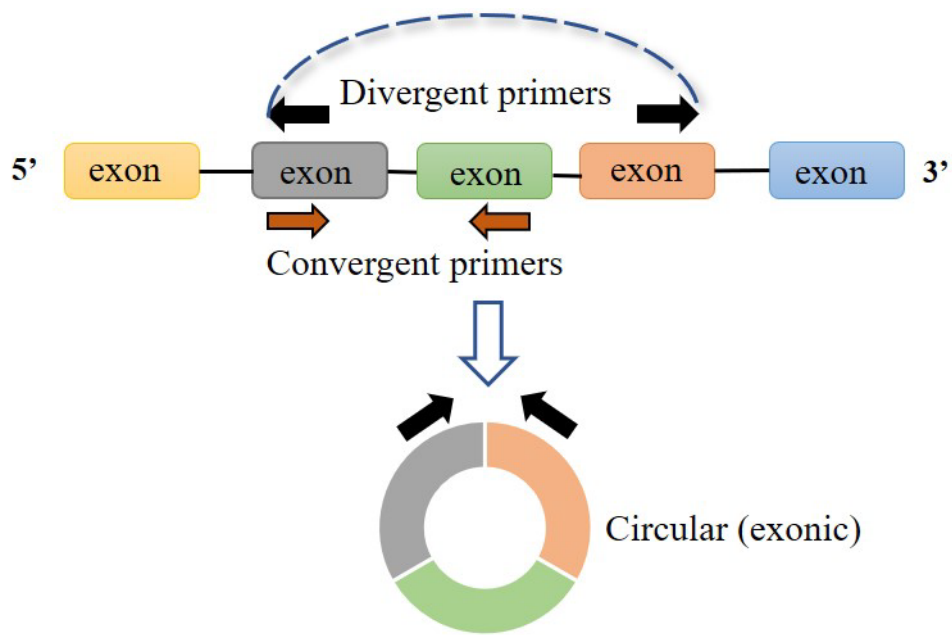

Supplementary Figure 2: Schematic of the divergent primers used for the detection of cicrRNAs.

Supplement: Supplementary file 2 — Additional file 2: Figure S2. Schematic of the divergent primers used for the detection of cicrRNAs. [file 13054_2020_3146_MOESM2_ESM.pdf]
