# Supplementary material for: The circular RNA landscape in specific peripheral blood mononuclear cells of critically ill patients with sepsis
Source: Crit Care. 2020 Jul 13;24:423. doi: 10.1186/s13054-020-03146-4 (PMC7359566; doi:10.1186/s13054-020-03146-4)
Supplement: Supplementary file 4 — Additional file 4: Figure S3. Linear RNA transcriptional changes in monocytes, CD4+ and CD8+ T-cells and CD19+ B-cells of CAP patients compared to healthy subjects. [file 13054_2020_3146_MOESM4_ESM.pdf]

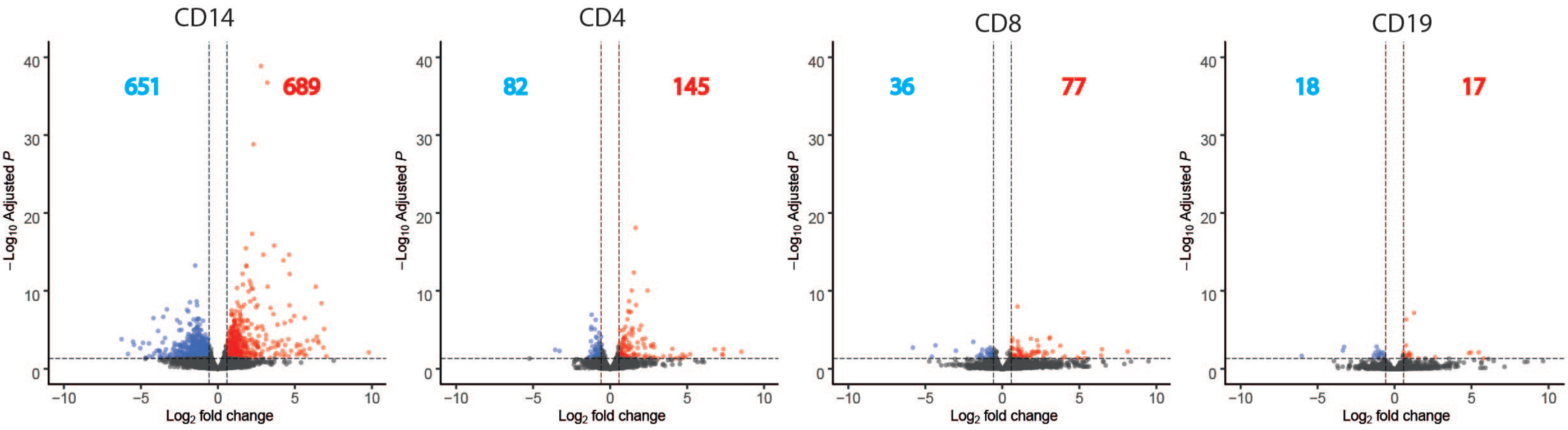

Supplementary Figure 3: Volcano plot (integrating log2 fold changes and multiple comparison adjusted p-values) representing the transcriptional changes that occur in monocytes, CD4 and CD8 T-cells and B-cells compared to healthy. Numbers in top left and right corners indicate number of genes with significantly differential expression, upregulated (red) or downregulated (blue). Horizontal line represents the multiple-test adjusted significance threshold ($p<0.01$) and vertical line represents the log fold change ($FC=\pm0.58$). Grey dots shows the genes that were not significantly overexpressed or underexpressed according to this threshold
